# Psychological and physiological effects of extended DMT

**DOI:** 10.1177/02698811231196877

**Published:** 2023-10-28

**Authors:** Lisa X Luan, Emma Eckernäs, Michael Ashton, Fernando E Rosas, Malin V Uthaug, Alexander Bartha, Samantha Jagger, Kiara Gascon-Perai, Lauren Gomes, David J Nutt, David Erritzøe, Robin L Carhart-Harris, Christopher Timmermann

**Affiliations:** 1 Centre for Psychedelic Research, Division of Psychiatry, Department of Brain Sciences, Imperial College London, London, UK; 2 Unit for Pharmacokinetics and Drug Metabolism, Department of Pharmacology, Sahlgrenska Academy at University of Gothenburg, Gothenburg, Sweden; 3 Centre for Complexity Science, Imperial College London, London, UK; 4 Department of Informatics, University of Sussex, Brighton, UK; 5 Centre for Eudaimonia and Human Flourishing, University of Oxford, Oxford, UK; 6 Department of Neuropsychology and Psychopharmacology, Faculty of Psychology and Neuroscience, Maastricht, Netherlands; 7 Somnivore Ply Ltd, Australia; 8 Psychedelics Division–Neuroscape, Department of Neurology, University of California San Francisco, San Francisco, CA, USA

**Keywords:** Psychedelics, dimethyltryptamine, serotonin, ayahuasca, consciousness

## Abstract

N,N-Dimethyltryptamine (DMT) is a serotonergic psychedelic that induces a rapid and transient altered state of consciousness when inhaled or injected via bolus administration. Its marked and novel subjective effects make DMT a powerful tool for the neuroscientific study of consciousness and preliminary results show its potential role in treating mental health conditions. In a within-subjects, placebo-controlled study, we investigated a novel method of DMT administration involving a bolus injection paired with a constant-rate infusion, with the goal of extending the DMT experience. Pharmacokinetic parameters of DMT estimated from plasma data of a previous study of bolus intravenous DMT were used to derive dose regimens necessary to keep subjects in steady levels of immersion into the DMT experience over an extended period of 30 min, and four dose regimens consisting of a bolus loading dose and a slow-rate infusion were tested in eleven healthy volunteers (seven male, four female, mean age ± SD = 37.09 ± 8.93 years). The present method is effective for extending the DMT experience in a stable and tolerable fashion. While subjective effects were maintained over the period of active infusion, anxiety ratings remained low and heart rate habituated within 15 min, indicating psychological and physiological safety of extended DMT. Plasma DMT concentrations increased consistently starting 10 min into DMT administration, whereas psychological effects plateaued into the desired steady state, suggesting the development of acute psychological tolerance to DMT. Taken together, these findings demonstrate the safety and effectiveness of continuous IV DMT administration, laying the groundwork for the further development of this method of administration for basic and clinical research.

## Introduction

N,N-Dimethyltryptamine (DMT) is a naturally occurring psychedelic with a mechanism of action linked to agonism at the 5-HT2A receptor (Nichols, 2016). It can induce a transient and immersive altered state of consciousness, characterized by complex and vivid visual imagery, as well as somatosensory, affective and cognitive effects (Gouzoulis-Mayfrank et al., 2005; Strassman and Qualls, 1994). Perhaps most distinctive to the experience is the frequently reported sense of immersion into what is perceived to be another world or dimension. This experience is twinned with reports of encountering ‘entities’ or ‘sentient presences’ in about half of cases (Davis et al., 2020; Lawrence et al., 2022; Michael et al., 2021; Strassman, 1995; Timmermann et al., 2019). The DMT experience shares similarities with the near-death experience (Timmermann et al., 2018) and is frequently deemed profound, at times leading to lasting revisions of beliefs about the nature of reality and consciousness (Timmermann et al., 2021). DMT is therefore a powerful tool for the study of consciousness. The subjective experience of immersion into a richly content-full experience, while feeling decoupled from the immediate environmental sensorium, makes DMT a unique compound for the study of human consciousness, and previous neuroimaging studies have significantly advanced our understanding of its effects on the human brain (Acosta-Urquidi, 2015; Alamia et al., 2020; Pallavicini et al., 2020; Timmermann et al., 2019, 2023). Additionally, DMT has gained increased interest for its potential therapeutic benefits in treating mental health disorders such as depression (D’Souza et al., 2022; *SPL026 (DMT Fumarate) in Healthy Subjects and MDD Patient*s; Timmermann, 2020).

When ingested orally, DMT is rapidly metabolized in the gastrointestinal system by monoamine oxidase (MAO) (Riba et al., 2015) which renders it psychologically inactive. In both recreational and ritual settings, DMT is commonly given in the form of ayahuasca (Domínguez-Clavé et al., 2016), a brew containing DMT and harmala alkaloids that inhibit MAO (Riba et al., 2015), which upon oral ingestion, leads to alterations in consciousness lasting for 4–6 h. Alternatively, DMT can be inhaled (vaping/smoking) (Winstock et al., 2014), or administered parenterally (e.g., intramuscularly, or intravenously as a bolus). These forms of administration result in a very short duration of subjective effects: when administered via bolus intravenous (IV) injection, DMT’s subjective effects begin within seconds, reaching their peak intensity within 2–3 min and subsiding thereafter, with negligible effects felt after about 30 min (Strassman, 1996; Timmermann et al., 2019). IV DMT administration allows for precise control over dosing, which makes it the preferred route of administration for investigating its effects in humans (Gouzoulis-Mayfrank et al., 2005; Strassman and Qualls, 1994; Timmermann et al., 2019, 2023). DMT appears to be physiologically safe at effective doses and repeated administrations of bolus IV DMT do not produce any obvious psychological tolerance – for example, four doses of DMT have been safely administered in a single session apparently without diminished psychological effects (Strassman et al., 1996); this property, combined with the rapid onset, short time-course and specific character of its effects, makes DMT possibly unique among serotonergic psychedelics and suitable for continuous IV administration (Gallimore and Strassman, 2016).

The development of a prolonged DMT experience would be an important step towards allowing for a closer study of its phenomenology and neurobiological effects, and would make it possible to control the length, intensity, and dynamics of the DMT experience, with the potential for real-time adjustment. This could make extended DMT a valuable tool in conjunction with therapy, to tailor the psychedelic effects to the individual needs of each patient and their clinical condition, as well as for consciousness research, whereby the intensity of the DMT experience can be adjusted according to specific hypotheses and research questions. In a previous study, continuous infusions of DMT were safely administered for up to 90 min (Gouzoulis-Mayfrank et al., 2005) and the infusion rates were reported to be determined via a previous pilot study with six subjects. However, this report lacked information on how strong and sustained these effects were over time. Another approach was proposed, aiming to generate stable subjective effects via a continuous infusion of the drug (Gallimore and Strassman, 2016) but unfortunately, this protocol has not been implemented in human participants. Overall, no pharmacokinetically informed protocols of IV DMT have been tested in humans to date.

The present study developed the first systematic protocol for the continuous IV infusion of DMT and tested its effectiveness in healthy volunteers. The main goal of the study was to provide first steps towards establishing infusion parameters for maintaining a steady state of DMT effects for a chosen length of time.

## Materials and methods

### Study design

In a single-blind, placebo-controlled study using a within-subjects repeated measures design, 11 healthy volunteers received up to four different doses of bolus plus slow-rate infusions of DMT over 30 min. Dosing sessions were separated by at least 2 weeks. On dosing visits, EEG activity as well as subjective and autonomic effects were measured acutely and retrospectively, and blood samples were collected for pharmacokinetic purposes (EEG findings and pharmacokinetic modelling will be published elsewhere). The study was approved by the National Research Ethics Service Committee London – Brent and the Health Research Authority and was conducted in accordance with the revised Declaration of Helsinki (2013), the International Committee on Harmonization Guidelines in Good Clinical Practice, and the National Health Service Research Governance Framework. The study was sponsored and approved by Imperial College London’s Joint Research and Compliance Office and the National Institute for Health Research/Wellcome Trust Imperial Clinical Research Facility gave site-specific approval for the study. The study was conducted under a Home Office licence for research with Schedule 1 drugs.

### Dosing schedule

Different dose regimens were simulated based on a separate population pharmacokinetic reanalysis of data from a previous study (Strassman and Qualls, 1994), yielding new two-compartment model parameter estimates (unpublished). Four dose levels of a combination of an IV loading bolus dose followed by a constant-rate infusion were tested, with the aim to induce and sustain moderate-to-high intensity of effects over 30 min, while maintaining psychological safety (i.e., low levels of anxiety). The bolus IV injection was delivered over 30 s, followed by a saline flush for 15 s. The constant-rate infusion was started 1 min after the beginning of the bolus injection and lasted 29 min, for a total of 30 min of DMT administration (i.e., bolus plus constant-rate infusion). Table 1 shows the doses administered to subjects in this study. To ensure safety, a pilot session involved the first participant receiving a dose of 1.5 mg bolus plus 0.2 mg/min of DMT fumarate. The data for this dose is not included here as it was not repeated across participants, induced negligible psychological effects, and was not intended for analyses. The following infusions initially followed the lowest calculated dose regimen, with the first subject receiving a bolus dose of 6 mg of DMT fumarate, followed by a constant rate infusion of 0.63 mg/min, corresponding to projected low doses according to previous research (Strassman and Qualls, 1994). Doses were increased for subsequent dosing sessions if the previous dose was well-tolerated and produced an average peak subjective intensity of < 7.5 (scale 0–10), with the aim of identifying a dose that sustains a high level of intensity throughout the infusion to be used in future studies investigating the phenomenology and brain activity related to states of immersion commonly reported for DMT.

**Table 1.** Dosing schedule of continuous IV infusions of DMT. Doses are expressed for DMT fumarate. A bolus injection was given over 30 s, followed by a saline flush over 15 s. The continuous slow-rate infusion was started 1 min after the beginning of the bolus injection and maintained for 29 min.

Doses	Bolus (mg)	Inf (mg/min)	Total (mg)	Participants (*n*)
Dose 1	6	0.63	24.19	6
Dose 2	10	1.05	40.32	10
Dose 3	14	1.46	56.45	9
Dose 4	18	1.88	72.58	6

### Study drugs

On dosing days, DMT fumarate (Cole Parmer Instrument Company Limited, St Neots, Cambridgeshire, United Kingdom; Onyx Scientific, Sunderland, Tyne & Wear, United Kingdom) was reconstituted with saline to give a 5 mg/ml solution and sterile-filtered into sterile vials. To keep infusion rates equal across doses, DMT was drawn into syringes for the bolus and slow-rate infusion, then saline was added to create a total of 5 ml and 15 ml, respectively. The bolus injection and saline flush were administered by a study physician, and the constant-rate infusion was delivered with a BD Alaris GH plus syringe pump (BD Switzerland Sàrl, Eysins, Vaud, Switzerland), with the infusion rate set to 30 ml/h.

### Participants

Eleven healthy participants (seven male, four female, mean age ± SD = 37.09 ± 8.93 years, range = 26–51 years) took part in this study. Participants were recruited via word of mouth. Exclusion criteria for study participation were age <18, current or previously diagnosed psychiatric disorder, family history of psychotic disorder, a medically significant condition which rendered them unsuitable for the study (e.g., heart condition, diabetes), an abnormal physical exam, electrocardiogram, or blood test, no prior experience with a psychedelic drug, previous adverse response to a hallucinogenic drug, blood or needle phobia, a positive pregnancy test at screening or during the study, and excessive use of alcohol or other drugs (>40 units per week). Participants were asked to not consume any alcoholic drinks 48 h before each study visit, and to not consume any illicit drugs 2 weeks before their first study visit until 2 weeks after their last study visit. Drug of abuse tests and pregnancy tests (where applicable) were performed before each dosing visit. All subjects gave written informed consent in accordance with the Declaration of Helsinki.

### Study procedures

All volunteers attended a screening visit to determine eligibility to be enrolled in the study. During this visit, a physical examination (weight, ECG, blood pressure, heart rate (HR) and neurological examination) and routine blood test were performed, as well as a psychiatric interview (see ‘Participants’ for exclusion criteria). Subsequently, volunteers provided informed consent to be enrolled in the study and completed questionnaires.

The dosing visits took place in a calm, decorated hospital room at Imperial College Research Facility. On dosing days, two research staff, one medic, and one research subject were present. Subjects were reminded of the study procedures and were asked to verbally provide re-consent if they wished to continue. Urine screens for drugs of abuse and pregnancy were performed. Participants were fitted with an EEG cap, an ECG was taken (to ensure safety), and cannulation took place on both arms. Blood samples were drawn from one cannula, and continuous infusions of DMT/placebo were administered via the other cannula. Participants were made comfortable in a semi-supine position and baseline EEG recordings were taken (eyes open and eyes closed). Shortly before dosing started, a body scan meditation was performed to help participants relax. During dosing, participants wore an eye mask and were instructed to keep their eyes closed. Low-volume ambient music (https://soundcloud.com/user-202717649-642667767/colour-field-94) was played through headphones to ensure psychological comfort. Dosing took place at around noon, during which DMT was administered for 30 min (see Table 1). EEG and acute subjective and autonomic effects were recorded for 8 min pre-dosing and 52 min post-dosing.

### Experience sampling of subjective effects

#### Intensity

Subjective intensity of effects was assessed acutely via experience sampling from 8 min prior to 52 min after the start of the bolus injection. For this, audio prompts were played through headphones, and participants were instructed to verbally give subjective ratings of the intensity of the experience (from 0 = ‘no effects’ to 10 = ‘most intense imaginable’). These were collected at −8, −6, −4, −2, 0, 1, 2, 3, 4, and every 2 min thereafter, with minute 0 being the start of the bolus injection.

#### Anxiety

Subjective ratings of anxiety were also collected every 4 min using a similar procedure (from 0 = ‘no anxiety’ to 10 = ‘most anxiety imaginable’) in order to assess the psychological safety of DMT infusions.

### Retrospective assessments of subjective effects

#### ASC and MEQ-30

Once the acute drug effects had subsided (~30 min following the end of administration), participants completed the altered states of consciousness (ASC) Scale and the Mystical Effects Questionnaire (MEQ-30). The ASC scale measures altered states of consciousness. The 94 items make up five dimensions (5D-ASC; ‘Oceanic boundlessness’, ‘Anxious ego dissolution’, ‘Visionary restructuration’, ‘Auditory alterations’, and ‘Reduced vigilance’; (Dittrich, 1998)), and 11 lower-order scales (11D-ASC; (Studerus et al., 2010)). The MEQ-30 assesses mystical-type experiences. The 30 items are sorted into four scale scores ‘Mystical’, ‘Positive mood’, ‘Transcendence of time and space’, and ‘Ineffability’ (Barrett et al., 2015).

#### Dynamic subjective effects

Additionally, participants were asked to retrospectively provide ratings of various dimensions of their experience over time (from 0 = ‘none/not at all’ to 10 = ‘an extreme amount/extremely’). These dimensions included ‘Immersion’, ‘Entity encounters’, ‘Ego dissolution’, ‘Visual imagery’, ‘Emotional experience’, ‘Auditory hallucinations’, ‘Bodily dissociation’, ‘Bodily sensations’, ‘Meaningfulness’, ‘Metacognition’, ‘Overall richness’, and ‘Visual richness’ over time. The definitions for each item can be found in Supplemental Table S1.

### Heart rate

HR was measured and monitored from 8 min before until 52 min after the beginning of the bolus injection with an E4 Empatica wristband (Empatica Srl, Milan, Italy) which participants wore on their wrist during the dosing period. The results reported here correspond to the recorded HR at 2-min intervals.

### Plasma DMT concentrations

Plasma levels of DMT were repeatedly assessed at baseline and 2, 5, 10, 20, 29, 32, 37, 40, 50, 60, 80, 100, 120, 150, and 180 min after the beginning of bolus injection of DMT. Blood samples (up to 6 ml) were collected into EDTA tubes, kept on wet ice, and centrifuged within 1h of collection. The harvested plasma was stored at −80°C before being shipped on dry ice to the University of Gothenburg for analysis. Samples were analysed according to a previously published liquid chromatography tandem mass spectrometry method (Eckernäs et al., 2022a). In brief, a methanolic solution containing internal standards, was added to plasma samples, followed by protein precipitation with acetonitrile. The samples were centrifuged, supernatants were transferred to new tubes and evaporated to dryness before reconstitution in aqueous mobile phase. Chromatography was performed using a diphenyl column with gradient elution (0.1% formic acid in methanol/water) and the mass spectrometer was operated in multiple reaction monitoring mode (Eckernäs et al., 2022a).

### Statistical analysis

Linear mixed-effects models (LMMs) were constructed to test the relationship between doses, timepoints, and the response variables of intensity, anxiety, HR, DMT plasma concentrations, ASC scores and MEQ-30 scores. LMMs exhibit several advantages over repeated measures Analyses of Variance (ANOVAs) in dealing with the present data: (1) LMMs control for the potential influence of random variables (i.e., subject-driven interindividual variability) by including them as random effects; (2) LMMs can cope with missing data; and (3) they are able to include grouping hierarchies such as the present partially crossed groups (i.e., each subject received multiple but not all DMT doses).

LMM analyses were run in R (version 4.2.2) using the *lme4* (Bates et al., 2015) package. Models included subjective intensity, anxiety, HR, plasma DMT concentration, ASC scores and MEQ-30 scores as the dependent variable, respectively. For all models, the participant ID was accounted for via a random intercept.

For variables that were measured at multiple timepoints (subjective intensity, anxiety, HR, and plasma DMT concentration), dose and timepoint were added as fixed effects, and the dependent variable was assessed via change scores from the placebo condition for the same subject at that given timepoint. For those models, both dose and time were treated as categorical variables, and the earliest timepoint (minute -8) of Dose 1 was specified as a reference. While the investigation of the differences between each pair of doses is of interest, the small sample size motivated us to reduce the number of comparisons by focusing the statistical analyses on contrasting higher doses against Dose 1. Consequently, we report the summary statistics of Dose 1 compared to placebo, and the added effects of higher doses compared to Dose 1. The resulting model for these outcome variables was specified as follows:

Outcomevariable~Dose+Timepoint+(1|Subject).

For variables that do not include a temporal dimension (5D-ASC and MEQ-30 scales), one LMM was fitted for each subscale. For these models, only Dose was added as a fixed effect, and analyses were performed on raw scores. The resulting model for these outcome variables was specified as follows:

Outcomevariable~Dose+(1|Subject).

For all mixed models, significance was estimated via the *lmerTest* package (Kuznetsova et al., 2017), and false discovery rate (FDR) correction was used to adjust for multiple comparisons (Benjamini and Hochberg, 1995), with significance established at *p* < 0.05.

No statistical analysis was performed on the dimensions of the experience over time, as these were assessed retrospectively and hence were susceptible to recall bias. They are reported qualitatively to inform future studies employing acute experience sampling to investigate the extended DMT experience.

## Results

**Figure 1.**
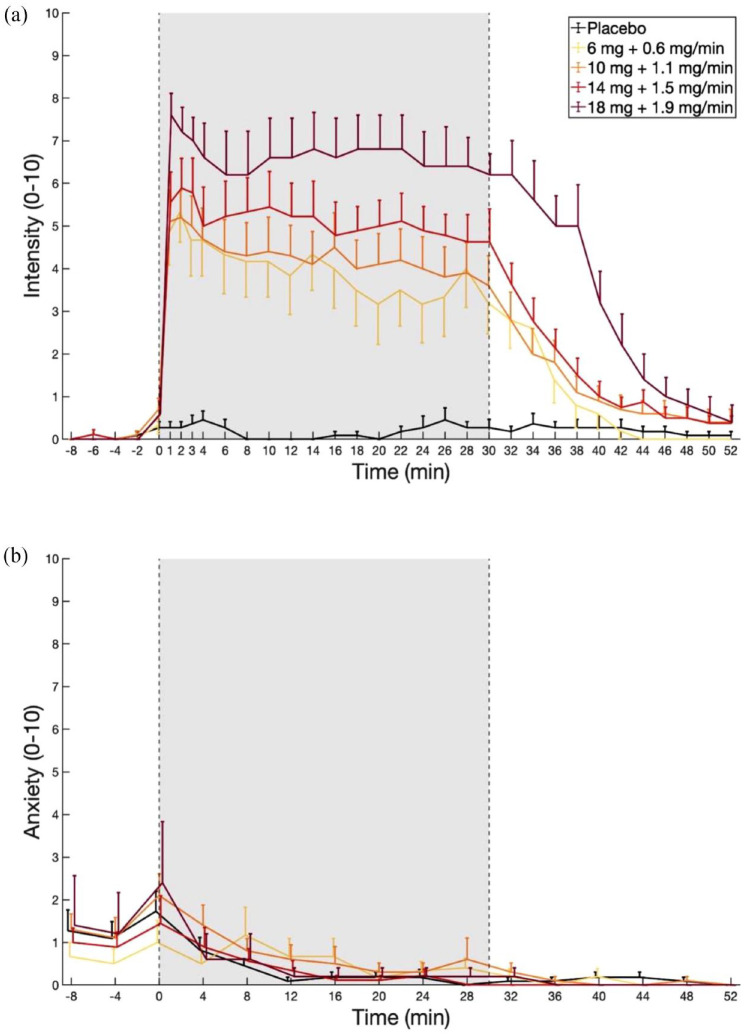
Experience sampling of subjective effects induced by continuous infusions of placebo and different doses of DMT. The bolus IV injection was administered at time 0. The constant-rate infusion started at min 1 and was maintained until minute 30. (a) Peak effects of DMT were successfully extended via continuous infusion. A significant dose-response relationship for Intensity was found for minutes 1–40 (assessed using a 0–10 scale with 0 = ‘no effects’ and 10 = ‘most intense effects imaginable’). (b) Anxiety ratings were given on a scale from 0 to 10, with 0 = ‘no anxiety’ and 10 = ‘most intense anxiety imaginable’. The data are expressed as the mean ± SEM.

In total, six participants received Dose 1, ten participants received Dose 2, nine participants received Dose 3, and five participants received Dose 4. Two participants reached the maximum amount of dosing sessions allowed by the protocol without reaching the goal average peak intensity of >7.5 (Participant 1 received a pilot dose (see above), and Dose 1 was repeated for Participant 3 due to a faulty infusion pump), and three participants dropped out without completing all dosing sessions. Due to the lack of differential effects seen between Dose 1 and Dose 2 (see Figure 1), Dose 1 was omitted from participant 7 onwards, to allow a focus on higher doses (i.e., Dose 4) while ensuring compliance with the study protocol. An overview of doses administered and any adverse reactions that occurred during and after dosing can be found in Table 2.

**Table 2.** Overview of participants, completed doses, adverse events, and data included in the analyses. On one occasion, the participant requested to terminate the continuous infusion at minute 26. Parameters for the doses are given in Table 1.

Participant	Doses received	Adverse events	Notes
1	1, 2, 3		Received an initial pilot dose of 1.5 mg bolus plus 0.2 mg/min of DMT (not reported here) and thus completed the four doses the protocol allowed and could not receive Dose 4
2	1, 2, 3	Dose 3: anxiety with chest discomfort (nothing abnormal was found on an ECG performed immediately after the infusion)	Dose 3 terminated at minute 26 upon request – all data up to minute 26 included in analysis
3	1, 2, 3		Dose 1 was repeated due to infusion line blocking on the first attempt and thus completed the four doses the protocol allowed and could not receive Dose 4
4	1, 2, 3, 4		
5	1, 2		Dropped out after Dose 2 due to personal reasons not related to adverse events
6	1		Infusion line blocked at minute 27 and 29 on Dose 1 – all data up to minute 26 included in analysis Dropped out after Dose 1 due to personal reasons not related to adverse events
7	2, 3, 4		
8	2, 3, 4	Dose 4: slight nausea	
9	2, 3, 4		Infusion line blocked on Dose 4 – excluded from analysis
10	2, 3, 4		
11	2, 3, 4		

*Note*: Participant 2 experienced anxiety and chest pains on Dose 3 starting from approx. minute 20 and requested to terminate the infusion at minute 26. Anxiety and chest pains subsided within 10 min of the end of infusion, and follow-up examinations (ECG, blood tests) did not show any abnormalities, suggesting that chest pains were of a psychogenic nature. Dose 1 was omitted for participants 7–11 to prioritise higher doses (Dose 2, 3, 4).

### Experience sampling of subjective effects

#### Intensity

Substantial increases in intensity ratings were observed with all tested doses, as shown in Figure 1. Using a bolus IV injection paired with a constant-rate infusion, participants were maintained at relatively stable levels of drug effects over the period of infusion. The onset of subjective effects was quick, occurring within the first minute of the beginning of the bolus IV injection being administered. After an initial peak, the subjective effects remained relatively stable throughout the duration of the continuous infusion, with the plateau of effects stabilising at a slightly lower level than the initial peak (Figure 1(a)). LMM analysis showed the temporal profile of the effect, revealing at first a significant increase in intensity for Dose 1 compared to placebo at minute 1 (*β* = 5.33, *t* = 15.5, *p* < 0.001) which lasted until minute 42 (*β* = 0.83, *t* = 2.35, *p* = 0.026), with peak intensity reached at minute 2 (*β* = 5.50, *t* = 15.9, *p* < 0.001). Additionally, other doses exhibited significant average increases when compared with the changes observed in Dose 1 (Dose 2: *β* = 1.09, *t* = 7.93, *p* < 0.001; Dose 3: *β* = 1.85, *t* = 12.7, *p* < 0.001; Dose 4: *β* = 3.12, *t* = 17.91, *p* < 0.001), resulting in a significant dose-effect relationship throughout the whole 30 min infusion and for 12 min following the end of infusion (*p* < 0.05, FDR corrected for multiple comparisons). Summary statistics can be found in Supplemental Table S2.

#### Anxiety

Overall, participants experienced low levels of anxiety before and throughout dosing indicating that extended administration of DMT was well tolerated (Figure 1(b)). LMM analysis showed no significant increases in anxiety during the peak of the experience under Dose 1 with respect to placebo, and only showed significant differences at minutes 8 (*β* = 0.60, *t* = 2.99, *p* = 0.019), 12 (*β* = 0.70, *t* = 3.49, *p* = 0.008) and 28 (*β* = 0.69, *t* = 3.38, *p* = 0.008, all FDR corrected) due to a quicker drop of the initial anxiety in the placebo condition. Moreover, the model revealed no significant effect associated to Dose. These results suggest that the initial increase in anxiety was related to psychological anticipation, rather than direct dose-dependent drug effects. Summary statistics can be found in Supplemental Table S3.

### Retrospective assessments of subjective effects

#### ASC and MEQ-30

**Figure 2.**
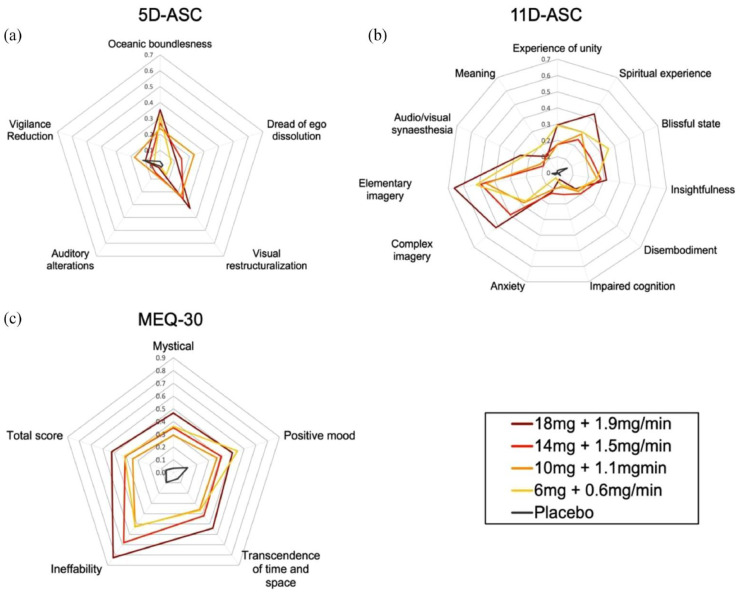
Retrospective assessment of subjective effects induced by continuous infusions of placebo and different doses of DMT. LMM analysis revealed: (a) a significant increase for all doses compared with placebo in ‘Oceanic boundlessness’ and ‘Visual restructuralization’, for Dose 2, 3 and 4 compared with placebo in ‘Dread of ego-dissolution’, and for Dose 2 and 3 compared with placebo in ‘Auditory alterations’, (b) significant increases for all doses compared with placebo in ‘Unity’, ‘Meaning’, ‘Spiritual experience’ ‘Blissful state’, ‘Insightfulness’, ‘Complex imagery’, and ‘Elementary imagery’, for Dose 3 compared with placebo in ‘Disembodiment’, and for Dose 2 and 3 compared with placebo in ‘Impaired cognition’, and (c) a significant increase for all doses versus placebo on the MEQ-30 total and all subscales. Summary statistics can be found in Supplemental Tables S4–S6. Scores are scaled between 0–1 and expressed as the mean.

LMM analysis revealed significant increases on the 5D-ASC subscales ‘Oceanic boundlessness’ and ‘Visual restructuralization’ for all doses compared with placebo. There were significant increases for Dose 2, 3 and 4 compared with placebo on the 5D-ASC subscale ‘Dread of ego-dissolution’, and for Dose 2 and 3 compared with placebo on the 5D-ASC subscale ‘Auditory alterations’. For the 11D-ASC, LMM analysis showed significant increases on the subscales ‘Unity’, ‘Meaning’, ‘Spiritual experience’ ‘Blissful state’, ‘Insightfulness’, ‘Complex imagery’, and ‘Elementary imagery’ for all doses compared with placebo. Additionally, there were significant increases for Dose 3 compared with placebo on the subscale ‘Disembodiment’, and significant increases for Dose 2 and 3 compared with placebo on the subscale ‘Impaired cognition’. No significant differences between any dose and placebo were found for the subscales ‘Anxiety’ and ‘Audio/visual synaesthesia’. Lastly, LMM analysis revealed significant increases in the MEQ-30 total as well as all subscales for all doses with respect to placebo. Results are shown in Figure 2, and summary statistics can be found in Supplemental Tables S4–S6.

#### Dynamic subjective effects

**Figure 3.**
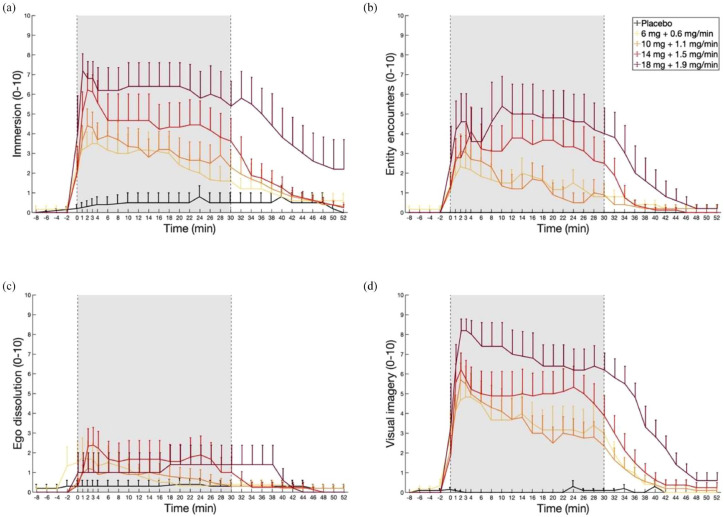
Retrospective assessment of ‘Immersion’, ‘Entity encounters’, ‘Ego dissolution’ and ‘Visual imagery’ over continuous infusions of placebo and different doses of DMT (minutes 0–30). DMT infusions induced sustained effects of ‘Immersion’, ‘Entity encounters’ and ‘Visual imagery’, while ‘Ego-dissolution’ remained low across doses. The data are expressed as the mean ± SEM. Additional results can be found in Supplemental Figures S1–S8.

Retrospective assessment of different dimensions of the experience showed that participants’ experience of ‘Immersion’ and ‘Visual imagery’ largely followed subjective intensity scores over time, whereas ‘Entity encounters’ increased during the latter part of the infusion on Doses 3 and 4. Participants experienced minimal ‘Ego dissolution’ following the DMT infusions in this study (Figure 3). Additionally, participants’ experience of ‘Bodily sensations’, ‘Visual richness’ and ‘Overall richness’ of the experience also largely followed subjective intensity scores over time, while the experience of ‘Bodily sensations’, ‘Emotional experience’, and ‘Meaningfulness’ of the experience increased during the latter part of the infusion and experiences of ‘Auditory hallucinations’ and ‘Bodily dissociation’ remained low. Additional results can be found in Supplemental Figures S1–S8.

### Heart rate

**Figure 4.**
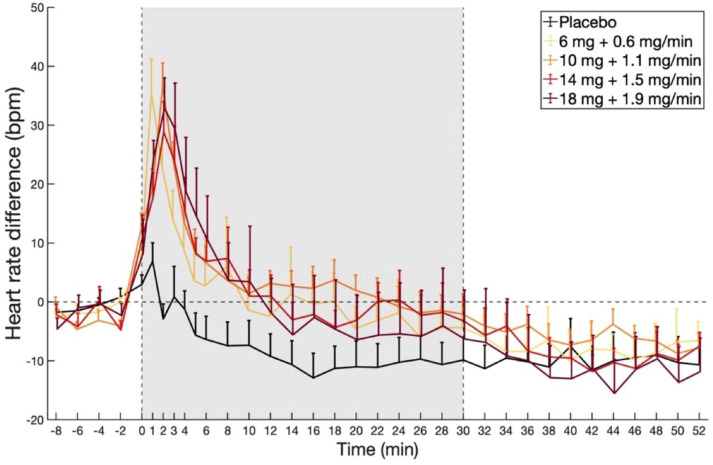
Acute changes in HR following continuous infusions of placebo and different doses of DMT (minutes 0–30). While the initial period of DMT administration is associated with an elevated HR, this then returns to baseline levels and stabilises. Values are expressed as average differences in beats per minute (bpm) from baseline HR, defined as the average HR between minutes -8 and 0, ± SEM.

Changes in HR following DMT dosing with respect to placebo are shown in Figure 4. LMM analysis showed significant increases in HR for Dose 1 compared to placebo, starting at minute 0 (*β* = 8.29, *t* = 2.75, *p* = 0.013), peaking at minute 2 (*β* = 35.5, *t* = 11.8, *p* < 0.001), and lasting until minute 24 (*β* = 8.95, *t* = 2.97, *p* = 0.007). Additionally, there were added effects of Dose, which however, were only significant after FDR correction for Dose 3 (*β* = 4.28, *t* = 3.22, *p* = 0.003) and Dose 4 (*β* = 8.67, *t* = 5.24, *p* < 0.001). Summary statistics can be found in Supplemental Table S7. Overall, these findings reveal that HR increases induced by continuous DMT stabilise following the bolus period, while subjective effects remain elevated suggesting physiological safety.

### Plasma DMT concentrations

**Figure 5.**
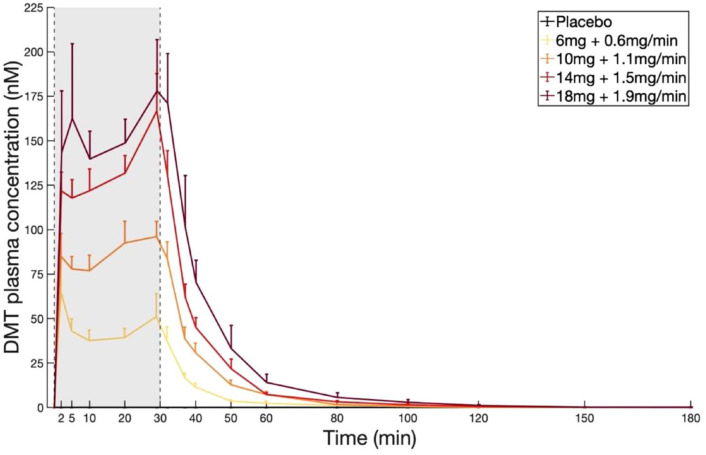
DMT plasma concentrations after administration of DMT fumarate IV bolus dose followed by constant-rate infusion at four dose levels (minutes 0–30). Plasma concentrations peak after bolus IV injection and before the end of the continuous IV infusion. There was a significant dose-effect relationship for all doses, for all measured timepoints until minute 50. The data are expressed as the mean ± SEM.

Plasma concentrations of DMT over time for all doses of DMT are shown in Figure 5. LMM analysis showed an increase in plasma concentrations of DMT for Dose 1 compared to placebo, which remained significant for all timepoints following bolus injection until minute 50, exhibiting a first peak at minute 2 (*β* = 103.4, *t* = 14.2, *p* < 0.001) and a second peak at minute 29 (*β* = 124.2, *t* = 17.1, *p* < 0.001) – the latter coinciding with the last blood sampling timepoint before the end of the infusion. On average, peak concentrations at the end of infusion were slightly higher than those achieved by the bolus dose. Moreover, results show added effects for all higher doses compared to Dose 1 (Dose 2: *β* = 11.7, *t* = 2.53, *p* = 0.019; Dose 3: *β* = 35.2, *t* = 8.10, *p* < 0.001; Dose 4: *β* = 45.8, *t* = 9.01, *p* < 0.001), resulting in a significant dose-effect relationship for all doses, for all measured timepoints until minute 50 (all FDR corrected for multiple comparisons). Following the end of the infusion, plasma concentrations of DMT decreased quickly, reaching <1 nM 90 min after the end of the infusion. Summary statistics can be found in Supplemental Table S8. The increase in plasma levels of DMT from minutes 10 to 30, while subjective effects remain sustained suggest the development of short-term psychological tolerance induced by DMT.

## Discussion

The present dose-response study tested a novel methodology designed to extend the typically transient DMT experience via continuous IV infusion. This was combined with a bolus loading dose aiming to achieve prolonged effects within a short time span. Our results showed that IV infusions of DMT – at doses ranging from 6 mg + 0.6 mg/min to 18 mg + 1.9 mg/min – were psychologically and physiologically well tolerated, and that the protocol was effective at extending the duration of the psychological effects of DMT, achieving a steady-state of subjective effects, with low-to-negligible changes in anxiety. Subjective drug effects appeared within 1 min of the start of the bolus injection, peaked at 2 min, and settled at a slightly lower-than-peak intensity for the duration of the infusion. Subjective drug effects decreased in intensity shortly after the end of infusion, resolving almost entirely 20 min after the end of infusion. Intensity of the drug effects increased dose-dependently. Ratings of anxiety generally remained low during the duration of the infusion, with a transient increase in anxiety seen just around the start of DMT administration, suggesting psychological tolerability of the tested continuous infusion protocol. Significant differences between all DMT doses and placebo were found for all ASC subscales except ‘Dread of ego-dissolution’ and ‘Auditory alterations’ on the 5D-ASC, as well as, ‘Disembodiment’ and ‘Impaired cognition’ on the 11D-ASC, which showed significant increases only for the higher doses, and ‘Vigilance reduction’ on the 5D-ASC as well as ‘Anxiety’ and ‘Audio/visual synaesthesia’ on the 11D-ASC, which showed no significant differences. Conversely, there were significant increases between all DMT doses and placebo for the MEQ-30 total and all subscales. An initial peak in average plasma DMT concentrations was observed corresponding to the bolus IV injection. A second increase was observed corresponding to the continuous infusion, starting at minute 5–10 and continuing steadily until the end of infusion, at which point plasma levels dropped exponentially, reaching <1 nM within 90 min of the end of infusion. Plasma DMT concentrations increased dose-dependently. Finally, HR peaked quickly after the bolus IV injection, then exhibited significant decreases with respect to baseline within 10 min of the start of the bolus injection, suggesting it was in part anxiety-related and showing that longer infusions do not overload the autonomic system. This habituation of physiological effects while subjective effects remain elevated indicates physiological safety of extended DMT infusions and is consistent with previous findings showing the development of physiological tolerance following the administration of four closely spaced bolus injections of 20 mg of DMT fumarate (Strassman et al., 1996).

Importantly, although subjective ratings of intensity largely followed plasma concentrations of DMT during the first 20 min, a decoupling was observed from minute 20 onwards. While subjective intensity of drug effects decreased after an initial peak and remained stable with a slight downward slope for the remainder of the infusion, plasma levels of DMT generally increased throughout the infusion. These findings may suggest a progressive development of acute psychological tolerance to DMT during continuous infusion, a finding not seen in previous studies employing this compound. One potential explanation for the dissociation between subjective intensity and DMT plasma concentrations in the later part of the infusion is the development of psychological habituation to the effects over time, that is, effects of the same strength lose salience over time, whereas sharp transitions (i.e., as the one induced by the bolus), induce a stronger psychological response. However, the observed variability between individuals makes it difficult to draw any final conclusions based on this analysis. Future studies are needed to address the cause and extent of short-term tolerance induced by continuous DMT to better understand and develop infusion protocols that elicit desired subjective effects.

Based on the apparent reduction of sensory effects over time, a refined infusion protocol may be required to better pair the subjective effects achieved through the bolus injection of DMT with those maintained by the continuous infusion and avoid a decrease over time. Additionally, the slight mismatch between peak concentrations achieved at the end of infusion and those achieved after the bolus dose could likely be avoided with slightly adjusted combinations of bolus doses and infusion rates. At the conception of this study, DMT plasma concentration data was only available from one previous study using bolus IV injections of DMT (Strassman and Qualls, 1994). Therefore, the pharmacokinetic model used for this study has scope for improvement. Our team has since collected further plasma DMT data, including a previous study (Timmermann et al., 2019), which has been used to model the relationship between DMT pharmacokinetics and effects (Eckernäs et al., 2022b, 2023). This model can be further refined with the data reported here to improve our understanding of DMT pharmacokinetics and pharmacodynamics (PK-PD) and refine the dosing parameters used in future studies, to further enhance the stability of the extended DMT experience. This could be useful in neuroimaging studies with purposes related to consciousness research, for example, investigating the neural correlates of ‘entity experiences’ or ‘immersion into alternate realities’, which are commonly described distinctive features of the subjective experience induced by DMT (Davis et al., 2020; Lawrence et al., 2022; Michael et al., 2021; Strassman, 1995; Timmermann et al., 2019).

Importantly, inter-individual variability in dose-concentration response and dose-effects response was large. A roughly two-fold range was observed for both plasma concentrations and intensity ratings for a single dose. Although variability in dose-response of this size is not unique to DMT – similar effects are seen for anaesthetic substances administered via bolus injection (Schüttler and Ihmsen, 2000), and variances of 20–30% in plasma drug concentrations achieved using well-established infusion protocols are common (Coetzee et al., 1995), it remains relevant to determine the variables driving this variability, in order to reduce it. One way to solve issues of individual variability is with personalized dosing: Individually based PK-PD parameters could be derived from plasma concentrations acquired from an initial DMT administration, and these parameters could then be used to determine dose regimens which will be effective in inducing a stable, extended DMT experience in one individual.

Psychedelic therapy is increasingly showing transdiagnostic relevance for treating several mental health conditions (Andersen et al., 2021; Kočárová et al., 2021). Preliminary results suggest that DMT specifically shows efficacy for treating depression, and it is currently being trialled in a placebo-controlled study (*SPL026 (DMT Fumarate) in Healthy Subjects and MDD Patients*). Due to the short half-life and associated fast metabolism of DMT, all acute subjective effects resolve within minutes after stopping the infusion, thus making it arguably safer than prolonged psychedelic states induced by oral psilocybin, LSD or MDMA. DMT is therefore an attractive alternative psychedelic intervention. Furthermore, longer infusion times can be explored for therapeutic purposes using the present infusion method. Based on our findings on the safety and feasibility of continuous DMT administration, we foresee a potential for continuous DMT infusions to be used in precision psychiatry, for example, adjusting dosing parameters to suit individual cases and scenarios.

Psychedelics present an intriguing challenge in psychiatry as their psychological response is known to be highly variable and, at times, unpredictable. This unpredictability is usually accounted for by both dose and so-called non-pharmacological, or contextual variables, commonly described as (mind-) ‘set’ and ‘setting’ (Hartogsohn, 2016). The high variability of plasma DMT concentrations for a single dose in this study suggests that the subject-specific PK profile may significantly influence the psychological response as well. In the future, an approach tailored to the PK characteristics of the individual may thus be highly valuable to induce acute subjective effects that have been found to predict improvements in mental health outcomes in psychedelic experiences (Yaden and Griffiths, 2020). In this way, infusion parameters can be tailored to the specific characteristics of patient populations, treatment outcomes, and individual variables that bear relevance in psychedelic medicine, for example, personality traits (Aday et al., 2021), genetics, and brain function (Moujaes et al., 2022). Importantly, personalised infusion parameters can be leveraged beyond attaining a stable plateau of effects. For example, longer infusion times and dynamic infusion parameters can be meaningfully employed to alter the speed of the onset and offset of effects and the occurrence of ‘rest periods’, where, for example, subjective effects could be tapered for a period to provide a period of more sober reflection. This study is a meaningful step forward in that direction. Here, we employed a bolus plus slow infusion protocol, but in future protocols, a more gradual onset may be favoured to allow participants to ‘acclimatise’ to the DMT state.

This pilot study tested a novel method of DMT administration and assessed the stability and tolerability of different doses of the drug delivered via continuous IV infusions. DMT exhibits pharmacokinetic properties that make it suitable for continuous IV infusion, and this study demonstrated the safety and feasibility of this method of administration. Continuous IV infusions of DMT administered in doses of up to 18 mg + 1.9 mg/min were psychologically and physiologically well-tolerated in healthy, psychedelic-experienced volunteers. No significant acute or persistent adverse effects were observed. It is important to note some limitations of this study. Firstly, this study was conducted on a small sample of eleven participants. Due to the small number of resulting observations, we tested the effects of Dose 1 compared to placebo, and the added effects of higher doses compared to Dose 1. Dose-related effects between higher doses were only assessed in a qualitative fashion. Future studies including larger sample sizes may be able to investigate the differences between each pair of doses of interest. Secondly, due to safety considerations (i.e., participants receiving a maximum of four DMT doses) and dropouts, there was variability in doses administered, with not all participants receiving all doses investigated. Relatedly, although several of the dropouts occurred for personal reasons, it is not possible to rule out the possibility that participants did not wish to receive more doses. However, it is important to acknowledge that all tested doses seemed psychologically safe for most participants, as can be seen from ratings of anxiety and the adverse events presented in Table 2. Lastly, this study adopted a fixed-order design and thus order effects may have occurred. While our results are not consistent with the development of long-term tolerance to DMT, it is still possible that some order effects may have occurred due to psychological reasons (i.e., participants guessing the order of administration). Future studies could directly test the occurrence of order effects by asking participants to guess the order of doses administered.

This study lays the groundwork for further explorations with extended IV infusions of DMT. The extended DMT experience may be valuable to explore further the phenomenology, neurobiology, and clinical outcomes associated with this unique state of consciousness. Furthermore, the successful and flexible extension of DMT administration poses a significant opportunity for the application of DMT in clinical and therapeutic settings, where the length, strength and dynamics of the psychedelic experience can be adjusted according to the needs of the individual.

## Supplemental Material

sj-docx-1-jop-10.1177_02698811231196877 – Supplemental material for Psychological and physiological effects of extended DMT Click here for additional data file. Supplemental material, sj-docx-1-jop-10.1177_02698811231196877 for Psychological and physiological effects of extended DMT by Lisa X Luan, Emma Eckernäs, Michael Ashton, Fernando E Rosas, Malin V Uthaug, Alexander Bartha, Samantha Jagger, Kiara Gascon-Perai, Lauren Gomes, David J Nutt, David Erritzøe, Robin L Carhart-Harris and Christopher Timmermann in Journal of Psychopharmacology
