# Supplementary material for: Psychological and physiological effects of extended DMT
Source: J Psychopharmacol. 2023 Oct 28;38(1):56–67. doi: 10.1177/02698811231196877 (PMC10851633; doi:10.1177/02698811231196877)
Supplement: sj-docx-1-jop-10.1177_02698811231196877 – Supplemental material for Psychological and physiological effects of extended DMT [file sj-docx-1-jop-10.1177_02698811231196877.docx]

Supplemental Material for:

**Psychological and physiological effects of extended DMT**


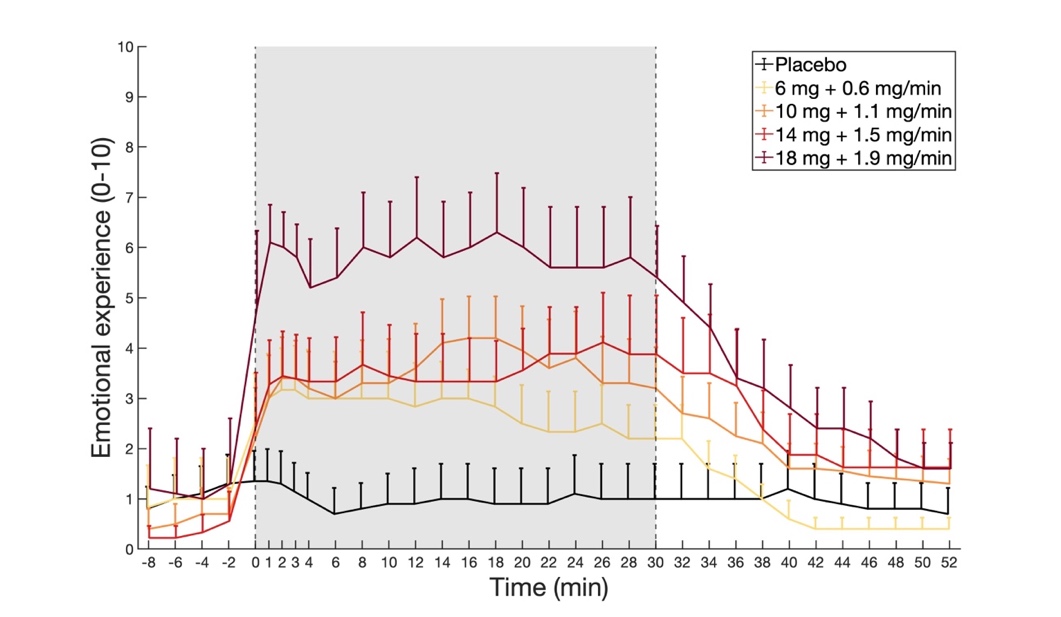


**Figure S1.** Subjective ratings of ‘Emotional experience’ over time following continuous infusions of placebo and different doses of DMT (minutes 0-30). Ratings were collected retrospectively after the end of the drug infusion. The data are expressed as the mean ± SEM.


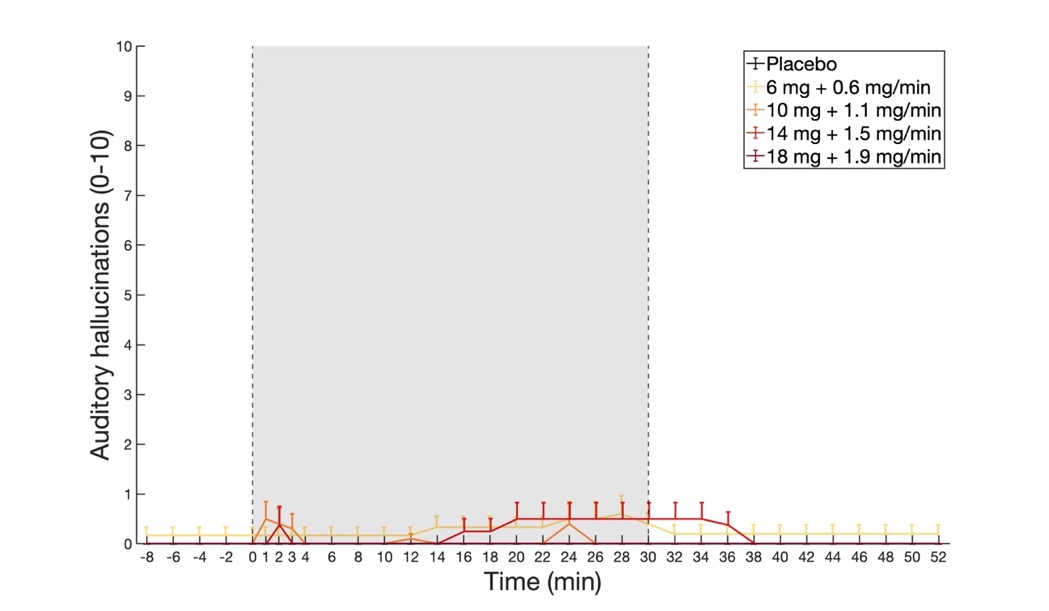


**Figure S2.** Subjective ratings of ‘Auditory hallucinations’ over time following continuous infusions of placebo and different doses of DMT (minutes 0-30). Ratings were collected retrospectively after the end of the drug infusion. The data are expressed as the mean ± SEM.


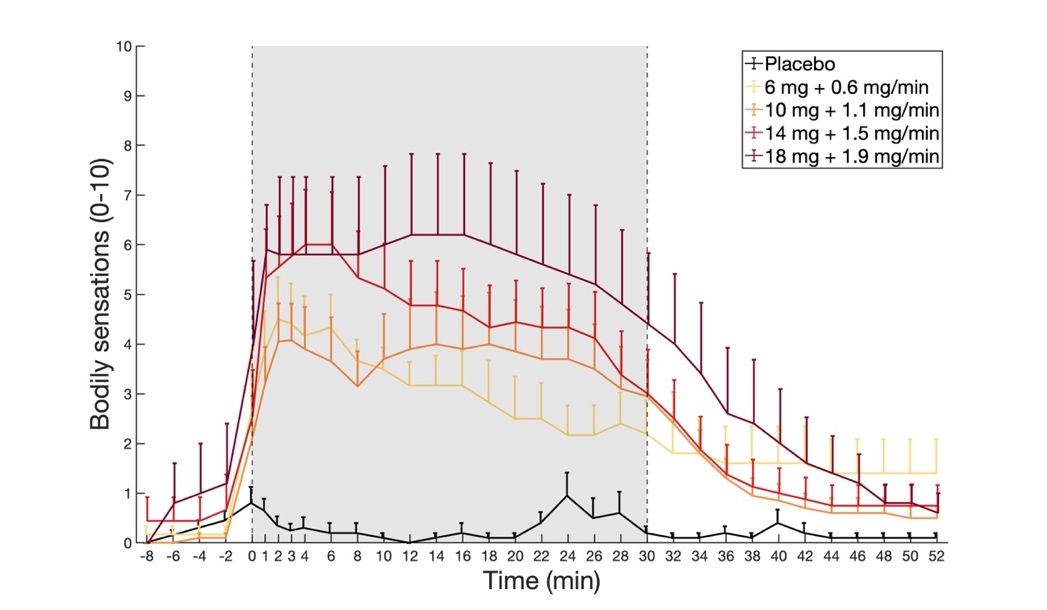


**Figure S3.** Subjective ratings of ‘Bodily sensations’ over time following continuous infusions of placebo and different doses of DMT (minutes 0-30). Ratings were collected retrospectively after the end of the drug infusion. The data are expressed as the mean ± SEM.


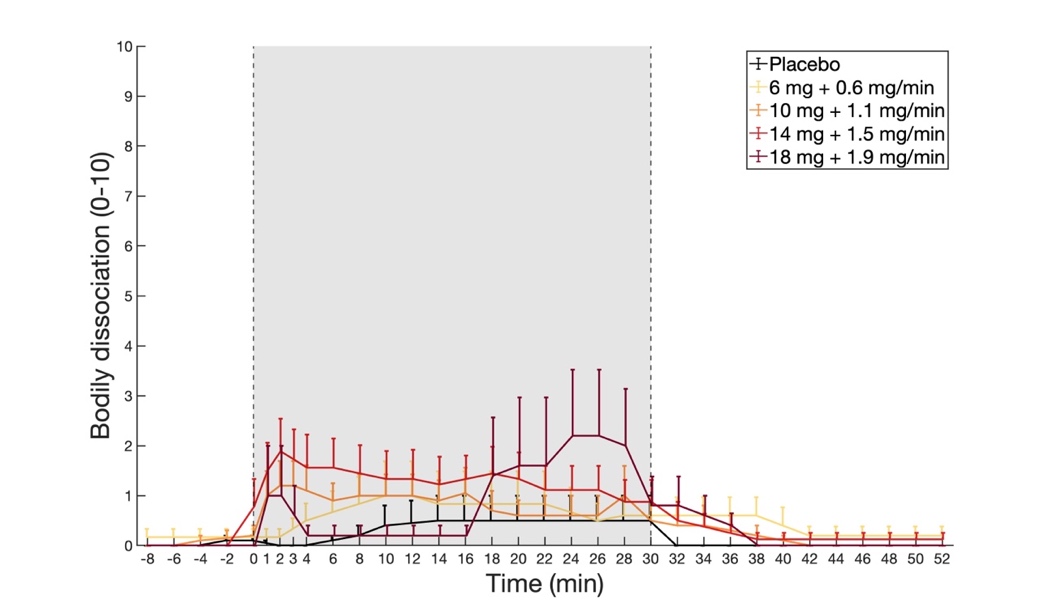


**Figure S4.** Subjective ratings of ‘Bodily dissociation’ over time following continuous infusions of placebo and different doses of DMT (minutes 0-30). Ratings were collected retrospectively after the end of the drug infusion. The data are expressed as the mean ± SEM.


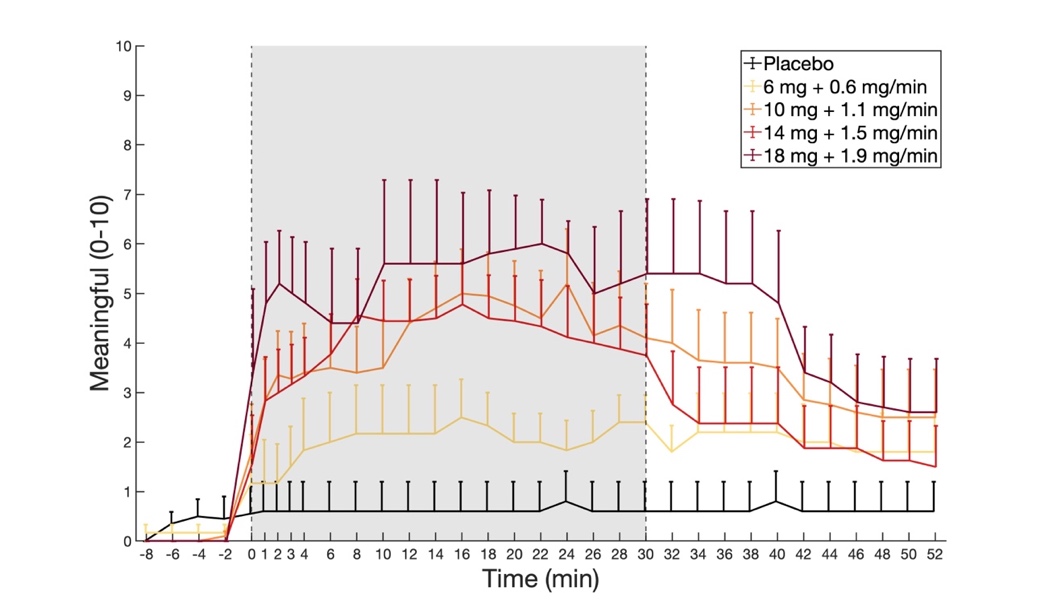


**Figure S5.** Subjective ratings of ‘Meaningfulness’ over time following continuous infusions of placebo and different doses of DMT (minutes 0-30). Ratings were collected retrospectively after the end of the drug infusion. The data are expressed as the mean ± SEM.


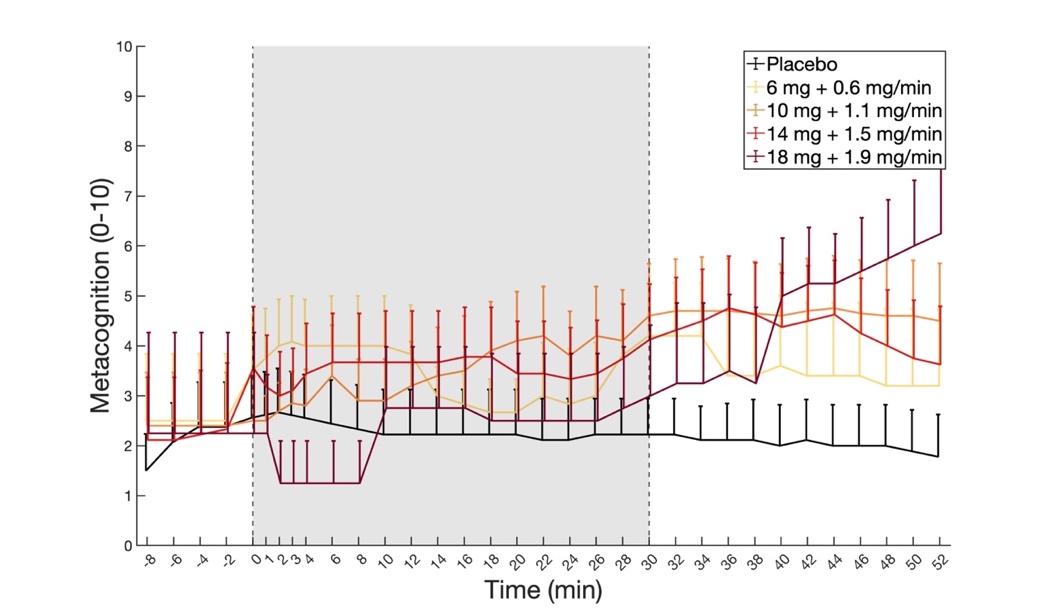


**Figure S6.** Subjective ratings of ‘Metacognition’ over time following continuous infusions of placebo and different doses of DMT (minutes 0-30). Ratings were collected retrospectively after the end of the drug infusion. The data are expressed as the mean ± SEM.


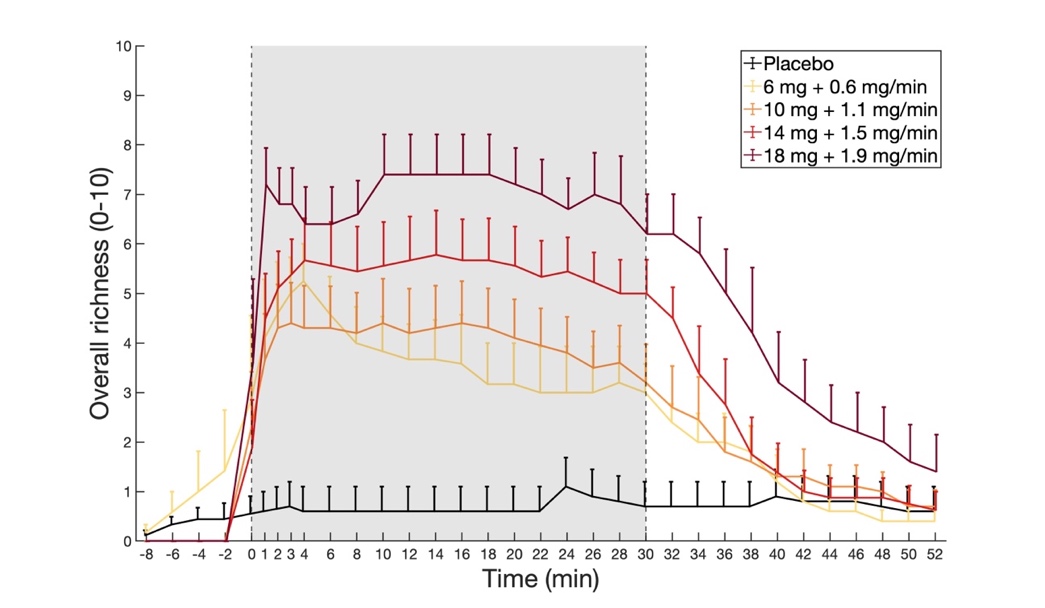


**Figure S7.** Subjective ratings of ‘Overall richness’ over time following continuous infusions of placebo and different doses of DMT (minutes 0-30). Ratings were collected retrospectively after the end of the drug infusion. The data are expressed as the mean ± SEM.


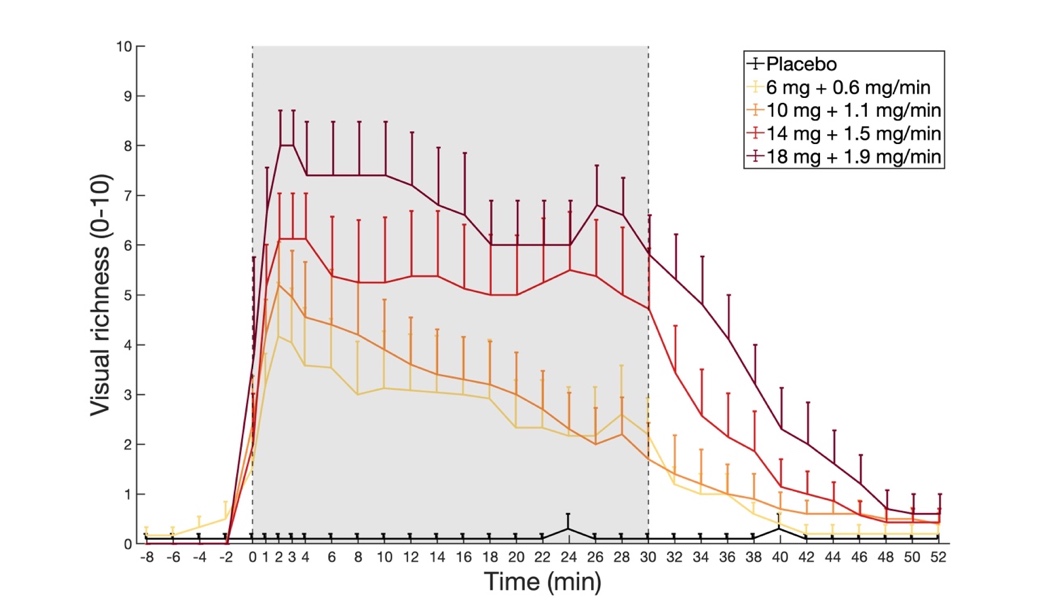


**Figure S8.** Subjective ratings of ‘Visual richness’ over time following continuous infusions of placebo and different doses of DMT (minutes 0-30). Ratings were collected retrospectively after the end of the drug infusion. The data are expressed as the mean ± SEM.

**Table S1.** Definitions for dynamic subjective effects.

| **Item** | **Definition** |
| --- | --- |
| Immersion | Feeling of being immersed or absorbed into a ‘space’ separate from the experimental environment |
| Entity encounters | Intensity of sensing a presence |
| Ego dissolution | Loosening of the distinction between the ‘self’ and the environment |
| Visual imagery | Perception of images with eyes closed |
| Emotional experience | Intensity of emotional content |
| Auditory hallucinations | Hearing sounds not originating from the experimental environment |
| Bodily dissociation | Losing awareness of the body |
| Bodily sensations | Effects felt in the body |
| Meaningful | How meaningful did the experience feel? |
| Metacognition | ‘Processing’ the experience |
| Overall ‘richness’ | How ‘rich’ did the experience feel in terms of richness of contents? |
| Visual ‘richness’ | How ‘rich’ did the visual experience feel in terms of richness of contents? |

**Table S2.** Summary table for the generalized linear mixed effects model of subjective intensity.

| **Random effects** | **σ^2^** | **SD** |  |  |
| --- | --- | --- | --- | --- |
| Subject intercept | 1.426 | 1.194 |  |  |
| Residual | 1.787 | 1.337 |  |  |
| **Fixed effects** | ***b*** | **SE** | ***t*** | ***p*** |
| Intercept | -1.454 | 0.446 | -3.258 | 0.005 ** |
| 10 mg + 1.1 mg/min | 1.087 | 0.137 | 7.933 | < 0.001 *** |
| 14 mg + 1.5 mg/min | 1.848 | 0.146 | 12.685 | < 0.001 *** |
| 18 mg + 1.9 mg/min | 3.119 | 0.174 | 17.910 | < 0.001 *** |
| Min -6 | 0.033 | 0.345 | 0.097 | 0.949 |
| Min -4 | 0.000 | 0.345 | 0.000 | 1.000 |
| Min -2 | -0.067 | 0.345 | -0.193 | 0.897 |
| Min 0 | 0.300 | 0.345 | 0.869 | 0.462 |
| Min 1 | 5.333 | 0.345 | 15.451 | < 0.001 *** |
| Min 2 | 5.500 | 0.345 | 15.934 | < 0.001 *** |
| Min 3 | 5.267 | 0.345 | 15.258 | < 0.001 *** |
| Min 4 | 4.767 | 0.345 | 13.809 | < 0.001 *** |
| Min 6 | 4.800 | 0.345 | 13.906 | < 0.001 *** |
| Min 8 | 4.900 | 0.345 | 14.196 | < 0.001 *** |
| Min 10 | 5.033 | 0.345 | 14.582 | < 0.001 *** |
| Min 12 | 4.867 | 0.345 | 14.099 | < 0.001 *** |
| Min 14 | 4.933 | 0.345 | 14.292 | < 0.001 *** |
| Min 16 | 4.700 | 0.345 | 13.616 | < 0.001 *** |
| Min 18 | 4.500 | 0.345 | 13.037 | < 0.001 *** |
| Min 20 | 4.633 | 0.345 | 13.423 | < 0.001 *** |
| Min 22 | 4.533 | 0.345 | 13.133 | < 0.001 *** |
| Min 24 | 4.100 | 0.345 | 11.878 | < 0.001 *** |
| Min 26 | 3.967 | 0.345 | 11.492 | < 0.001 *** |
| Min 28 | 4.290 | 0.351 | 12.205 | < 0.001 *** |
| Min 30 | 3.897 | 0.351 | 11.087 | < 0.001 *** |
| Min 32 | 3.361 | 0.351 | 9.563 | < 0.001 *** |
| Min 34 | 2.433 | 0.351 | 6.921 | < 0.001 *** |
| Min 36 | 2.111 | 0.351 | 6.007 | < 0.001 *** |
| Min 38 | 1.308 | 0.355 | 3.686 | < 0.001 *** |
| Min 40 | 0.825 | 0.351 | 2.348 | 0.026 * |
| Min 42 | 0.504 | 0.351 | 1.434 | 0.203 |
| Min 44 | 0.433 | 0.351 | 1.231 | 0.281 |
| Min 46 | 0.254 | 0.351 | 0.723 | 0.529 |
| Min 48 | 0.325 | 0.351 | 0.926 | 0.440 |
| Min 50 | 0.218 | 0.351 | 0.621 | 0.583 |
| Min 52 | 0.290 | 0.351 | 0.824 | 0.476 |

Note: Number of observations: 963, 11 subjects. P-values are FDR-corrected for multiple comparisons. Significance codes: p < 0.05 = *, p < 0.01 = **, p < 0.001 = ***.

**Table S3.** Summary table for the generalized linear mixed effects model of anxiety.

| **Random effects** | **σ^2^** | **SD** |  |  |
| --- | --- | --- | --- | --- |
| Subject intercept | 0.2958 | 0.5439 |  |  |
| Residual | 0.6044 | 0.7774 |  |  |
| **Fixed effects** | ***b*** | **SE** | ***t*** | ***p*** |
| Intercept | -0.312 | 0.232 | -1.341 | 0.316 |
| 10 mg + 1.1 mg/min | 0.106 | 0.113 | 0.938 | 0.473 |
| 14 mg + 1.5 m/min | -0.087 | 0.121 | -0.717 | 0.530 |
| 18 mg + 1.9 mg/min | -0.024 | 0.144 | -0.166 | 0.868 |
| Min -4 | 0.100 | 0.201 | 0.498 | 0.653 |
| Min 0 | 0.200 | 0.201 | 0.996 | 0.467 |
| Min 4 | 0.400 | 0.201 | 1.993 | 0.152 |
| Min 8 | 0.600 | 0.201 | 2.989 | 0.019 * |
| Min 12 | 0.700 | 0.201 | 3.487 | 0.008 ** |
| Min 16 | 0.467 | 0.201 | 2.325 | 0.098 |
| Min 20 | 0.300 | 0.201 | 1.495 | 0.276 |
| Min 24 | 0.333 | 0.201 | 1.661 | 0.232 |
| Min 28 | 0.691 | 0.204 | 3.380 | 0.008 ** |
| Min 32 | 0.405 | 0.204 | 1.983 | 0.152 |
| Min 36 | 0.263 | 0.204 | 1.284 | 0.316 |
| Min 40 | 0.155 | 0.204 | 0.760 | 0.530 |
| Min 44 | 0.155 | 0.204 | 0.760 | 0.530 |
| Min 48 | 0.298 | 0.204 | 1.459 | 0.276 |
| Min 52 | 0.370 | 0.204 | 1.808 | 0.193 |

Note: Number of observations: 466, 11 subjects. P-values are FDR-corrected for multiple comparisons. Significance codes: p < 0.05 = *, p < 0.01 = **, p < 0.001 = ***.

**Table S4.** Summary table for the linear mixed effects model of 5D-ASC subscales.

| Ocean | **Random effects** | **σ^2^** | **SD** |  | |  |
| --- | --- | --- | --- | --- | --- | --- |
|  | Subject intercept | 0.014 | 0.120 |  |  | |
|  | Residual | 0.015 | 0.122 |  |  | |
|  | **Fixed effects** | ***b*** | **SE** | ***t*** | ***p*** | |
|  | Intercept | 0.029 | 0.051 | 0.564 | 0.579 | |
|  | 6 mg + 0.6 mg/min | 0.253 | 0.064 | 3.953 | 0.001 ** | |
|  | 10 mg + 1.1 mg/min | 0.220 | 0.054 | 4.109 | 0.001 ** | |
|  | 14 mg + 1.5 m/min | 0.277 | 0.055 | 5.001 | < 0.001 *** | |
|  | 18 mg + 1.9 mg/min | 0.306 | 0.064 | 4.783 | < 0.001 *** | |
| DED | **Random effects** | **σ^2^** | **SD** |  |  | |
|  | Subject intercept | 0.002 | 0.044 |  |  | |
|  | Residual | 0.006 | 0.074 |  |  | |
|  | **Fixed effects** | ***b*** | **SE** | ***t*** | ***p*** | |
|  | Intercept | 0.017 | 0.026 | 0.666 | 0.510 | |
|  | 6 mg + 0.6 mg/min | 0.045 | 0.039 | 1.166 | 0.316 | |
|  | 10 mg + 1.1 mg/min | 0.090 | 0.033 | 2.765 | 0.025 * | |
|  | 14 mg + 1.5 m/min | 0.130 | 0.034 | 3.850 | 0.003 ** | |
|  | 18 mg + 1.9 mg/min | 0.090 | 0.039 | 2.322 | 0.045 * | |
| VRS | **Random effects** | **σ^2^** | **SD** |  |  | |
|  | Subject intercept | 0.007 | 0.084 |  |  | |
|  | Residual | 0.010 | 0.100 |  |  | |
|  | **Fixed effects** | ***b*** | **SE** | ***t*** | ***p*** | |
|  | Intercept | 0.011 | 0.039 | 0.286 | 0.777 | |
|  | 6 mg + 0.6 mg/min | 0.256 | 0.053 | 4.874 | < 0.001 *** | |
|  | 10 mg + 1.1 mg/min | 0.205 | 0.044 | 4.665 | < 0.001 *** | |
|  | 14 mg + 1.5 m/min | 0.253 | 0.046 | 5.556 | < 0.001 *** | |
|  | 18 mg + 1.9 mg/min | 0.276 | 0.053 | 5.246 | < 0.001 *** | |
| AUA | **Random effects** | **σ^2^** | **SD** |  |  | |
|  | Subject intercept | 0.001 | 0.025 |  |  | |
|  | Residual | 0.001 | 0.029 |  |  | |
|  | **Fixed effects** | ***b*** | **SE** | ***t*** | ***p*** | |
|  | Intercept | 0.000 | 0.012 | 0.016 | 0.988 | |
|  | 6 mg + 0.6 mg/min | 0.034 | 0.015 | 2.235 | 0.056 | |
|  | 10 mg + 1.1 mg/min | 0.039 | 0.013 | 2.992 | 0.014 * | |
|  | 14 mg + 1.5 m/min | 0.053 | 0.013 | 3.934 | 0.003 ** | |
|  | 18 mg + 1.9 mg/min | 0.032 | 0.015 | 2.095 | 0.056 | |
| VIR | **Random effects** | **σ^2^** | **SD** |  |  | |
|  | Subject intercept | 0.005 | 0.072 |  |  | |
|  | Residual | 0.003 | 0.057 |  |  | |
|  | **Fixed effects** | ***b*** | **SE** | ***t*** | ***p*** | |
|  | Intercept | 0.117 | 0.028 | 4.228 | 0.002 ** | |
|  | 6 mg + 0.6 mg/min | -0.021 | 0.030 | -0.694 | 0.493 | |
|  | 10 mg + 1.1 mg/min | -0.028 | 0.025 | -1.103 | 0.350 | |
|  | 14 mg + 1.5 m/min | -0.047 | 0.026 | -1.782 | 0.214 | |
|  | 18 mg + 1.9 mg/min | -0.038 | 0.030 | -1.238 | 0.350 | |

Note: ‘Ocean’ = ‘Oceanic boundlessness’, ‘VRS’ = ‘Visual restructuralization’, ‘AUA’ = ‘Auditory alterations’, ‘VIR’ = ‘Vigilance reduction’. Number of observations per subscale: 42, 11 subjects. P-values are FDR-corrected for multiple comparisons. Significance codes: p < 0.05 = *, p < 0.01 = **, p < 0.001 = ***.

**Table S5.** Summary table for the linear mixed effects model of 11D-ASC subscales.

| Unity | **Random effects** | **σ^2^** | **SD** |  | |  |
| --- | --- | --- | --- | --- | --- | --- |
|  | Subject intercept | 0.020 | 0.140 |  |  | |
|  | Residual | 0.025 | 0.159 |  |  | |
|  | **Fixed effects** | ***b*** | **SE** | ***t*** | ***p*** | |
|  | Intercept | 0.014 | 0.064 | 0.217 | 0.830 | |
|  | 6 mg + 0.6 mg/min | 0.229 | 0.083 | 2.748 | 0.020 * | |
|  | 10 mg + 1.1 mg/min | 0.169 | 0.070 | 2.424 | 0.028 * | |
|  | 14 mg + 1.5 m/min | 0.195 | 0.072 | 2.694 | 0.020 * | |
|  | 18 mg + 1.9 mg/min | 0.287 | 0.089 | 3.212 | 0.017 * | |
| Spiritual | **Random effects** | **σ^2^** | **SD** |  |  | |
|  | Subject intercept | 0.053 | 0.230 |  |  | |
|  | Residual | 0.017 | 0.132 |  |  | |
|  | **Fixed effects** | ***b*** | **SE** | ***t*** | ***p*** | |
|  | Intercept | 0.021 | 0.066 | 0.320 | 0.752 | |
|  | 6 mg + 0.6 mg/min | 0.243 | 0.079 | 3.068 | 0.006 ** | |
|  | 10 mg + 1.1 mg/min | 0.271 | 0.066 | 4.085 | 0.001 ** | |
|  | 14 mg + 1.5 m/min | 0.264 | 0.069 | 3.837 | 0.001 ** | |
|  | 18 mg + 1.9 mg/min | 0.404 | 0.085 | 4.747 | < 0.001 *** | |
| Blissful state | **Random effects** | **σ^2^** | **SD** |  |  | |
|  | Subject intercept | 0.042 | 0.204 |  |  | |
|  | Residual | 0.032 | 0.179 |  |  | |
|  | **Fixed effects** | ***b*** | **SE** | ***t*** | ***p*** | |
|  | Intercept | 0.069 | 0.082 | 0.843 | 0.411 | |
|  | 6 mg + 0.6 mg/min | 0.237 | 0.095 | 2.505 | 0.032 * | |
|  | 10 mg + 1.1 mg/min | 0.178 | 0.079 | 2.254 | 0.042 * | |
|  | 14 mg + 1.5 m/min | 0.232 | 0.082 | 2.835 | 0.027 * | |
|  | 18 mg + 1.9 mg/min | 0.279 | 0.102 | 2.752 | 0.027 * | |
| Insightfulness | **Random effects** | **σ^2^** | **SD** |  |  | |
|  | Subject intercept | 0.006 | 0.080 |  |  | |
|  | Residual | 0.016 | 0.127 |  |  | |
|  | **Fixed effects** | ***b*** | **SE** | ***t*** | ***p*** | |
|  | Intercept | 0.009 | 0.045 | 0.195 | 0.847 | |
|  | 6 mg + 0.6 mg/min | 0.235 | 0.066 | 3.564 | 0.002 ** | |
|  | 10 mg + 1.1 mg/min | 0.245 | 0.056 | 4.397 | < 0.001 *** | |
|  | 14 mg + 1.5 m/min | 0.281 | 0.058 | 4.879 | < 0.001 *** | |
|  | 18 mg + 1.9 mg/min | 0.293 | 0.071 | 4.151 | < 0.001 *** | |
| Disembodiment | **Random effects** | **σ^2^** | **SD** |  |  | |
|  | Subject intercept | 0.012 | 0.109 |  |  | |
|  | Residual | 0.020 | 0.140 |  |  | |
|  | **Fixed effects** | ***b*** | **SE** | ***t*** | ***p*** | |
|  | Intercept | 0.027 | 0.054 | 0.496 | 0.624 | |
|  | 6 mg + 0.6 mg/min | 0.105 | 0.073 | 1.438 | 0.202 | |
|  | 10 mg + 1.1 mg/min | 0.146 | 0.062 | 2.381 | 0.062 | |
|  | 14 mg + 1.5 m/min | 0.188 | 0.064 | 2.950 | 0.033 * | |
|  | 18 mg + 1.9 mg/min | 0.147 | 0.078 | 1.872 | 0.120 | |
| Impaired cog. | **Random effects** | **σ^2^** | **SD** |  |  | |
|  | Subject intercept | 0.002 | 0.044 |  |  | |
|  | Residual | 0.004 | 0.061 |  |  | |
|  | **Fixed effects** | ***b*** | **SE** | ***t*** | ***p*** | |
|  | Intercept | 0.004 | 0.023 | 0.181 | 0.858 | |
|  | 6 mg + 0.6 mg/min | 0.057 | 0.032 | 1.809 | 0.135 | |
|  | 10 mg + 1.1 mg/min | 0.085 | 0.027 | 3.184 | 0.009 ** | |
|  | 14 mg + 1.5 m/min | 0.135 | 0.028 | 4.882 | < 0.001 *** | |
|  | 18 mg + 1.9 mg/min | 0.054 | 0.034 | 1.600 | 0.151 | |
| Anxiety | **Random effects** | **σ^2^** | **SD** |  |  | |
|  | Subject intercept | 0.003 | 0.056 |  |  | |
|  | Residual | 0.012 | 0.111 |  |  | |
|  | **Fixed effects** | ***b*** | **SE** | ***t*** | ***p*** | |
|  | Intercept | 0.004 | 0.038 | 0.104 | 0.918 | |
|  | 6 mg + 0.6 mg/min | 0.014 | 0.058 | 0.239 | 0.918 | |
|  | 10 mg + 1.1 mg/min | 0.103 | 0.049 | 2.109 | 0.100 | |
|  | 14 mg + 1.5 m/min | 0.127 | 0.050 | 2.520 | 0.089 | |
|  | 18 mg + 1.9 mg/min | 0.120 | 0.062 | 1.954 | 0.100 | |
| Complex imag. | **Random effects** | **σ^2^** | **SD** |  |  | |
|  | Subject intercept | 0.013 | 0.114 |  |  | |
|  | Residual | 0.032 | 0.178 |  |  | |
|  | **Fixed effects** | ***b*** | **SE** | ***t*** | ***p*** | |
|  | Intercept | 0.018 | 0.064 | 0.281 | 0.781 | |
|  | 6 mg + 0.6 mg/min | 0.263 | 0.093 | 2.832 | 0.011 * | |
|  | 10 mg + 1.1 mg/min | 0.269 | 0.078 | 3.449 | 0.003 ** | |
|  | 14 mg + 1.5 m/min | 0.382 | 0.081 | 4.733 | < 0.001 *** | |
|  | 18 mg + 1.9 mg/min | 0.455 | 0.099 | 4.588 | < 0.001 *** | |
| Element. imag. | **Random effects** | **σ^2^** | **SD** |  |  | |
|  | Subject intercept | 0.023 | 0.150 |  |  | |
|  | Residual | 0.035 | 0.187 |  |  | |
|  | **Fixed effects** | ***b*** | **SE** | ***t*** | ***p*** | |
|  | Intercept | 0.035 | 0.072 | 0.483 | 0.633 | |
|  | 6 mg + 0.6 mg/min | 0.496 | 0.098 | 5.068 | < 0.001 *** | |
|  | 10 mg + 1.1 mg/min | 0.434 | 0.082 | 5.275 | < 0.001 *** | |
|  | 14 mg + 1.5 m/min | 0.478 | 0.085 | 5.614 | < 0.001 *** | |
|  | 18 mg + 1.9 mg/min | 0.595 | 0.105 | 5.665 | < 0.001 *** | |
| Audio/vis. syn. | **Random effects** | **σ^2^** | **SD** |  |  | |
|  | Subject intercept | 0.009 | 0.092 |  |  | |
|  | Residual | 0.037 | 0.192 |  |  | |
|  | **Fixed effects** | ***b*** | **SE** | ***t*** | ***p*** | |
|  | Intercept | 0.000 | 0.064 | 0.000 | 1.000 | |
|  | 6 mg + 0.6 mg/min | 0.251 | 0.100 | 2.517 | 0.066 | |
|  | 10 mg + 1.1 mg/min | 0.130 | 0.084 | 1.546 | 0.223 | |
|  | 14 mg + 1.5 m/min | 0.102 | 0.087 | 1.168 | 0.316 | |
|  | 18 mg + 1.9 mg/min | 0.248 | 0.106 | 2.335 | 0.066 | |
| Meaning | **Random effects** | **σ^2^** | **SD** |  |  | |
|  | Subject intercept | 0.005 | 0.069 |  |  | |
|  | Residual | 0.012 | 0.107 |  |  | |
|  | **Fixed effects** | ***b*** | **SE** | ***t*** | ***p*** | |
|  | Intercept | 0.004 | 0.038 | 0.102 | 0.920 | |
|  | 6 mg + 0.6 mg/min | 0.177 | 0.056 | 3.163 | 0.016 * | |
|  | 10 mg + 1.1 mg/min | 0.104 | 0.047 | 2.213 | 0.047 * | |
|  | 14 mg + 1.5 m/min | 0.145 | 0.049 | 2.978 | 0.016 * | |
|  | 18 mg + 1.9 mg/min | 0.131 | 0.060 | 2.189 | 0.047 * | |
|  |  |  |  |  |  | |

Note: ‘Unity’ = “Experience of unity’, ‘Spiritual’ = ‘Spiritual experience’, ‘Impaired cog.’ = ‘Impaired cognition’, ‘Complex imag.’ = ‘Complex imagery’, ‘Elementary imag.’ = ‘Elementary imagery’, ‘Audio/visual syn.’ = ‘Audio/visual synaesthesia’. Number of observations per subscale: 41, 11 subjects. P-values are FDR-corrected for multiple comparisons. Significance codes: p < 0.05 = *, p < 0.01 = **, p < 0.001 = ***.

**Table S6.** Summary table for the linear mixed effects model of MEQ-30 subscales.

| Mystical | **Random effects** | **σ^2^** | **SD** |  | |  |
| --- | --- | --- | --- | --- | --- | --- |
|  | Subject intercept | 0.039 | 0.198 |  |  | |
|  | Residual | 0.026 | 0.162 |  |  | |
|  | **Fixed effects** | ***b*** | **SE** | ***t*** | ***p*** | |
|  | Intercept | 0.033 | 0.077 | 0.423 | 0.677 | |
|  | 6 mg + 0.6 mg/min | 0.258 | 0.086 | 3.017 | 0.007 ** | |
|  | 10 mg + 1.1 mg/min | 0.280 | 0.071 | 3.924 | 0.001 ** | |
|  | 14 mg + 1.5 m/min | 0.373 | 0.074 | 5.042 | < 0.001 *** | |
|  | 18 mg + 1.9 mg/min | 0.433 | 0.092 | 4.713 | < 0.001 *** | |
| Positive mood | **Random effects** | **σ^2^** | **SD** |  |  | |
|  | Subject intercept | 0.025 | 0.157 |  |  | |
|  | Residual | 0.024 | 0.154 |  |  | |
|  | **Fixed effects** | ***b*** | **SE** | ***t*** | ***p*** | |
|  | Intercept | 0.121 | 0.066 | 1.825 | 0.084 | |
|  | 6 mg + 0.6 mg/min | 0.399 | 0.081 | 4.910 | 0.000 *** | |
|  | 10 mg + 1.1 mg/min | 0.278 | 0.068 | 4.088 | 0.001 *** | |
|  | 14 mg + 1.5 m/min | 0.336 | 0.070 | 4.770 | < 0.001 *** | |
|  | 18 mg + 1.9 mg/min | 0.370 | 0.087 | 4.245 | < 0.001 *** | |
| Transcendence | **Random effects** | **σ^2^** | **SD** |  |  | |
|  | Subject intercept | 0.011 | 0.107 |  |  | |
|  | Residual | 0.023 | 0.151 |  |  | |
|  | **Fixed effects** | ***b*** | **SE** | ***t*** | ***p*** | |
|  | Intercept | 0.061 | 0.056 | 1.086 | 0.288 | |
|  | 6 mg + 0.6 mg/min | 0.280 | 0.079 | 3.550 | 0.002 ** | |
|  | 10 mg + 1.1 mg/min | 0.314 | 0.066 | 4.730 | < 0.001 *** | |
|  | 14 mg + 1.5 m/min | 0.388 | 0.069 | 5.651 | < 0.001 *** | |
|  | 18 mg + 1.9 mg/min | 0.481 | 0.084 | 5.699 | < 0.001 *** | |
| Ineffability | **Random effects** | **σ^2^** | **SD** |  |  | |
|  | Subject intercept | 0.018 | 0.135 |  |  | |
|  | Residual | 0.033 | 0.182 |  |  | |
|  | **Fixed effects** | ***b*** | **SE** | ***t*** | ***p*** | |
|  | Intercept | 0.097 | 0.068 | 1.419 | 0.169 | |
|  | 6 mg + 0.6 mg/min | 0.400 | 0.095 | 4.202 | < 0.001 *** | |
|  | 10 mg + 1.1 mg/min | 0.451 | 0.080 | 5.633 | < 0.001 *** | |
|  | 14 mg + 1.5 m/min | 0.625 | 0.083 | 7.548 | < 0.001 *** | |
|  | 18 mg + 1.9 mg/min | 0.746 | 0.102 | 7.315 | < 0.001 *** | |
| Total score | **Random effects** | **σ^2^** | **SD** |  |  | |
|  | Subject intercept | 0.026 | 0.160 |  |  | |
|  | Residual | 0.017 | 0.132 |  |  | |
|  | **Fixed effects** | ***b*** | **SE** | ***t*** | ***p*** | |
|  | Intercept | 0.062 | 0.062 | 0.999 | 0.331 | |
|  | 6 mg + 0.6 mg/min | 0.300 | 0.070 | 4.301 | < 0.001 *** | |
|  | 10 mg + 1.1 mg/min | 0.306 | 0.058 | 5.265 | < 0.001 *** | |
|  | 14 mg + 1.5 m/min | 0.399 | 0.060 | 6.610 | < 0.001 *** | |
|  | 18 mg + 1.9 mg/min | 0.463 | 0.075 | 6.193 | < 0.001 *** | |

Note: ‘Transcendence’ = ‘Transcendence of time and space’. Number of observations per subscale: 41, 11 subjects. P-values are FDR-corrected for multiple comparisons. Significance codes: p < 0.05 = *, p < 0.01 = **, p < 0.001 = ***.

**Table S7.** Summary table for the generalized linear mixed effects model of heart rate.

| **Random effects** | **σ^2^** | **SD** |  |  |
| --- | --- | --- | --- | --- |
| Subject intercept | 52.09 | 7.217 |  |  |
| Residual | 113.89 | 10.672 |  |  |
| **Fixed effects** | ***b*** | **SE** | ***t*** | ***p*** |
| Intercept | 3.543 | 3.257 | 1.088 | 0.404 |
| 10 mg + 1.1 mg/min | 2.461 | 1.245 | 1.976 | 0.085 |
| 14 mg + 1.5 m/min | 4.281 | 1.330 | 3.219 | 0.003 ** |
| 18 mg + 1.9 mg/min | 8.671 | 1.655 | 5.239 | < 0.001 *** |
| Min -6 | -2.164 | 3.018 | -0.717 | 0.531 |
| Min -4 | -0.894 | 3.018 | -0.296 | 0.825 |
| Min -2 | -4.073 | 3.018 | -1.349 | 0.263 |
| Min 0 | 8.294 | 3.018 | 2.748 | 0.013 * |
| Min 1 | 16.542 | 3.018 | 5.480 | < 0.001 *** |
| Min 2 | 35.548 | 3.018 | 11.777 | < 0.001 *** |
| Min 3 | 23.650 | 3.018 | 7.835 | < 0.001 *** |
| Min 4 | 16.437 | 3.018 | 5.446 | < 0.001 *** |
| Min 5 | 16.075 | 3.018 | 5.326 | < 0.001 *** |
| Min 6 | 14.344 | 3.018 | 4.752 | < 0.001 *** |
| Min 8 | 14.520 | 3.018 | 4.810 | < 0.001 *** |
| Min 10 | 10.358 | 3.018 | 3.431 | 0.002 ** |
| Min 12 | 11.464 | 3.018 | 3.798 | < 0.001 *** |
| Min 14 | 11.457 | 3.018 | 3.796 | < 0.001 *** |
| Min 16 | 13.057 | 3.018 | 4.326 | < 0.001 *** |
| Min 18 | 11.447 | 3.018 | 3.792 | < 0.001 *** |
| Min 20 | 8.811 | 3.018 | 2.919 | 0.008 ** |
| Min 22 | 10.559 | 3.050 | 3.462 | 0.002 ** |
| Min 24 | 8.949 | 3.018 | 2.965 | 0.007 ** |
| Min 26 | 6.279 | 3.018 | 2.080 | 0.070 |
| Min 28 | 7.853 | 3.121 | 2.516 | 0.024 * |
| Min 30 | 5.632 | 3.161 | 1.782 | 0.126 |
| Min 32 | 5.288 | 3.121 | 1.694 | 0.146 |
| Min 34 | 3.208 | 3.121 | 1.028 | 0.417 |
| Min 36 | 3.034 | 3.121 | 0.972 | 0.423 |
| Min 38 | 2.876 | 3.121 | 0.921 | 0.440 |
| Min 40 | -0.204 | 3.121 | -0.065 | 0.965 |
| Min 42 | 4.384 | 3.121 | 1.404 | 0.248 |
| Min 44 | 3.052 | 3.121 | 0.978 | 0.423 |
| Min 46 | 0.872 | 3.121 | 0.279 | 0.825 |
| Min 48 | 2.330 | 3.121 | 0.746 | 0.527 |
| Min 50 | -0.137 | 3.121 | -0.044 | 0.965 |
| Min 52 | 2.462 | 3.121 | 0.789 | 0.514 |

Note: Number of observations: 809, 10 subjects. P-values are FDR-corrected for multiple comparisons. Significance codes: p < 0.05 = *, p < 0.01 = **, p < 0.001 = ***.

**Table S8.** Summary table for the generalized linear mixed effects model of plasma DMT concentrations.

| **Random effects** | **σ^2^** | **SD** |  |  |
| --- | --- | --- | --- | --- |
| Subject intercept | 127.1 | 11.27 |  |  |
| Residual | 895.4 | 29.92 |  |  |
| **Fixed effects** | ***b*** | **SE** | ***t*** | ***p*** |
| Intercept | -23.153 | 6.396 | -3.620 | 0.001 *** |
| 10 mg + 1.1 mg/min | 11.645 | 4.609 | 2.526 | 0.019 * |
| 14 mg + 1.5 m/min | 35.212 | 4.348 | 8.097 | < 0.001 *** |
| 18 mg + 1.9 mg/min | 45.755 | 5.079 | 9.008 | < 0.001 *** |
| Min 2 | 103.408 | 7.260 | 14.243 | < 0.001 *** |
| Min 5 | 98.786 | 7.260 | 13.606 | < 0.001 *** |
| Min 10 | 94.558 | 7.260 | 13.024 | < 0.001 *** |
| Min 20 | 104.186 | 7.260 | 14.350 | < 0.001 *** |
| Min 29 | 124.233 | 7.260 | 17.111 | < 0.001 *** |
| Min 32 | 105.050 | 7.340 | 14.311 | < 0.001 *** |
| Min 37 | 53.470 | 7.426 | 7.200 | < 0.001 *** |
| Min 40 | 38.383 | 7.340 | 5.229 | < 0.001 *** |
| Min 50 | 16.789 | 7.524 | 2.231 | 0.038 * |
| Min 60 | 6.810 | 7.524 | 0.905 | 0.497 |
| Min 80 | 2.137 | 7.524 | 0.284 | 0.969 |
| Min 100 | 0.743 | 7.524 | 0.099 | 0.969 |
| Min 120 | 0.296 | 7.623 | 0.039 | 0.969 |
| Min 150 | -0.583 | 7.524 | -0.077 | 0.969 |
| Min 180 | -0.667 | 7.524 | -0.089 | 0.969 |

Note: Number of observations: 438, 11 subjects. P-values are FDR-corrected for multiple comparisons. Significance codes: p < 0.05 = *, p < 0.01 = **, p < 0.001 = ***.
